# Proteomic analysis of *T. qataranse* exposed to lead (Pb) stress reveal new proteins with potential roles in Pb tolerance and detoxification mechanism

**DOI:** 10.3389/fpls.2022.1009756

**Published:** 2022-10-20

**Authors:** Kamal Usman, Serhiy Souchelnytskyi, Mohammad A. Al-Ghouti, Nabil Zouari, Mohammed H. Abu-Dieyeh

**Affiliations:** ^1^ Agricultural Research Station (ARS), Office of VP for Research & Graduate Studies, Doha, Qatar; ^2^ College of Medicine, Qatar University, Doha, Qatar; ^3^ Environmental Science Program, Department of Biological and Environmental Sciences, College of Arts and Science, Qatar University, Doha, Qatar; ^4^ Biological Science Program, Department of Biological and Environmental Sciences, College of Arts and Science, Qatar University, Doha, Qatar

**Keywords:** Tetraena qataranse, heavy metals, lead, proteomics, glycine rich proteins

## Abstract

Soil lead (Pb) contamination is one of the environmental problems facing the modern world. Sources of Pb in soil include industrial activities such as mining and smelting processes, agricultural activities such as application of insecticide and municipal sewage sludges, and urban activities such as use of lead in gasoline, paints, and other materials. Phytoremediation is the direct use of living green plants and is an effective, cheap, non-invasive, and environmentally friendly technique used to transfer or stabilize all the toxic metals and environmental pollutants in polluted soil or groundwater. Current work in this area is invested in elucidating mechanisms that underpin toxic-metal tolerance and detoxification mechanisms. The present study aims to gain insight into the mechanisms of Pb tolerance in *T. qataranse* by comparative proteomics. MALDI-TOF/MS and *in silico* proteome analysis showed differential protein expression between treated (50 mg kg^⎯1^ Pb) and untreated (0 mg kg^⎯1^ Pb) *T. qataranse.* A total of eighty-six (86) differentially expressed proteins, most of which function in ion and protein binding, antioxidant activity, transport, and abiotic response stress, were identified. In addition, essential stress-regulating metabolic pathways, including glutathione metabolism, cellular response to stress, and regulation of HSF1-mediated heat shock response, were also enriched. Also, at 52- and 49-kDa MW band areas, up to six hypothetical proteins with unknown functions were identified. Of these, protein AXX17_AT2G26660 is highly rich in glycine amino acid residues (up to 76%), suggesting that it is a probable glycine-rich protein (GRP) member. Although GRPs are known to be involved in plant defense against abiotic stress, including salinity and drought, there is no report on their role on Pb tolerance and or detoxification in plants. Further enrichment analysis in the current study reveals that the hypothetical proteins do not interact with known proteins and are not part of any enriched pathway. However, additional research is needed to functionally validate the role of the identified proteins in Pb detoxification mechanism.

## Introduction

In recent decades, rapid increases in urbanization and industrialization have caused the excessive release of heavy metals in farmlands with damaging effects on ecosystems (Nagajyoti et al., 2010; Alsafran et al., 2022). Among different heavy metals, lead (Pb) contamination in the soil lasts for 150–5,000 years and is hard to remediate, resulting in long-term accumulation in soil and organisms (Kanwal et al., 2014; Jiang et al., 2019). Plants do not require Pb for normal physiological and metabolic activities, and it significantly impairs plant growth and even results in death (Tang et al., 2020; Ghani et al., 2021). However, phytoremediation offers owners and managers of Pb-contaminated sites as an innovative and cost-effective option to address recalcitrant environmental contaminants (Parveen et al., 2020; Saleem et al., 2020c; Ahmad et al., 2022; Saleem et al., 2022). For metal contamination phytoremediation (and phytoextraction in particular), bioavailability of metals in contaminated soils is a crucial factor regulating metal uptake by plant roots (Saleem et al., 2020b; Bhantana et al., 2021). Current work in this area is invested in elucidating mechanisms that underpin toxic metal tolerance, bioaccumulation, and detoxification mechanisms to optimize plant systems for large-scale phytoremediation of polluted environments (Saleem et al., 2020a; Saleem et al., 2022). The phytoremediation of trace and heavy metals involves many physiological, biochemical, and molecular activities. In plants, metal-binding proteins, including phytochelatins (PCs) and metallothioneins (MTs), play essential roles in such mechanisms. PCs are induced by the activity of an enzyme, phytochelatin synthase (PCS), which is triggered by the activity of metal ions present (Cobbett, 2000; Sarma, 2011). While MTs are gene-encoded small cysteine-rich proteins (Kärenlampi et al., 2000), PCs are glutathione synthase products, and they bind to heavy metals, thereby forming a central part of the phyto-detoxification mechanism (Yurekli and Kucukbay, 2003; Fulekar et al., 2009).

Several studies demonstrate MT’s role in the protection of plants against the toxicity of heavy metals in soil, sediment, and water (Fulekar et al., 2009; Jabeen et al., 2009; Sheoran et al., 2010). According to Wu et al. (2010), MT’s and PC’s expression alongside organic acid synthesis together functions in heavy metal uptake by plants and their translocation to other tissue parts. Therefore, the expression of these natural chelators can be enhanced to increase the efficiency of heavy metal accumulation and translocation. However, it is a common consensus that even at the protein level, other unknown proteins or PCs and MTs may have vital roles in toxic metal bioaccumulation and detoxification in plants. Therefore, further efforts in elucidating the detoxification mechanisms are invested at characterizing and identifying new biomolecules involved in the transport and assist in vacuolar sequestration of toxic metals, including Pb (Seth, 2012; Ali et al., 2013).

Comparative proteomics provides excellent tools in this regard. Proteomics involves analyzing complex protein mixtures using various tools, specifically revealing information about individual proteins and their biological roles in living systems. Although the genome in the living system may maintain stability through many generations, protein populations may change during the development stage, especially under stress conditions, and these changes are not always proportionate (Kosová et al., 2011). Significant progress in the area of plant proteomics on model plants such as *Oryza sativa* and *Arabidopsis thaliana* led to improvement in the analysis of the plant proteome using high-throughput technologies, and today, proteins are automatically identified by sequence homology (Agrawal et al., 2012).

As a consequence of its complexity and dynamic nature, plant proteome analysis generally requires the use of different technologies (Baerenfaller et al., 2008). Some of the techniques are two-dimensional liquid chromatography matrix-assisted laser desorption/ionization time-of-flight mass spectrometry (2D-LC/TOF MS), liquid chromatography-tandem mass spectrometry (LC-MS/MS), and matrix-assisted laser desorption/ionization time-of-flight mass spectrometry (MALDI TOF/MS) (Haas et al., 2006; Xian et al., 2012). The power of these technologies led to increased interest in the proteomics study of plant hyperaccumulators acting in metal sequestration and detoxification (Visioli and Marmiroli, 2012). Recently, we have studied different levels of Pb (0, 25, 50, and 100 50 mg kg^⎯1^) in the field conditions and demonstrated that it is capable of hyperaccumulating Pb in greenhouse conditions when treated with 50 mg kg^⎯1^ Pb for 7 weeks (Usman et al., 2019). Although we have measured various morpho-physiological attributes of *T. qataranse* in our previous study, hypothetical proteins with unknown functions were still unknown in *T. qataranse* especially under the Pb stress. Therefore, the present study aims to gain insight into the mechanism of Pb tolerance in *T. qataranse* by comparative proteomics.

## Results

### The total proteins

The quality and quantity of proteins extracted from recalcitrant plant tissues are often limited by a high rate of contaminants, extraction buffer used, and overall sample preparation conditions. To optimize total protein extraction, samples were subjected to three extraction protocols, of which phenol/SDS with three prewash steps proved optimal, based on protein concentration (47 µg ml^⎯1^) and better electrophoretic separation (data not shown). Protein quantitation was performed using Bradford assay based on the protein bovine serum albumin (BSA) standard curve. Wang et al. (2006) noted that when extracting protein using phenol/SDS, some critical points must be noted: (i) samples should be kept at low temperature and (ii) the phenol phase following centrifugation should be carefully recovered (Wang et al., 2008); these were strictly observed in this study. Phenol/SDS buffers are vital in protein purification and separation and therefore critical to obtaining quality samples. Most of the time, the pH of the phenol solution was adjusted before use to meet the basic condition, where the distribution coefficients of proteins are usually greater than 100 (Pusztai, 1966). Buffered phenol solution (pH 8.0) and bromophenol blue used in this method were compatible with phenol with a pH indicator blue at greater than 7.0.

### Differentially expressed proteins due to Pb stress

**Figure 1:**
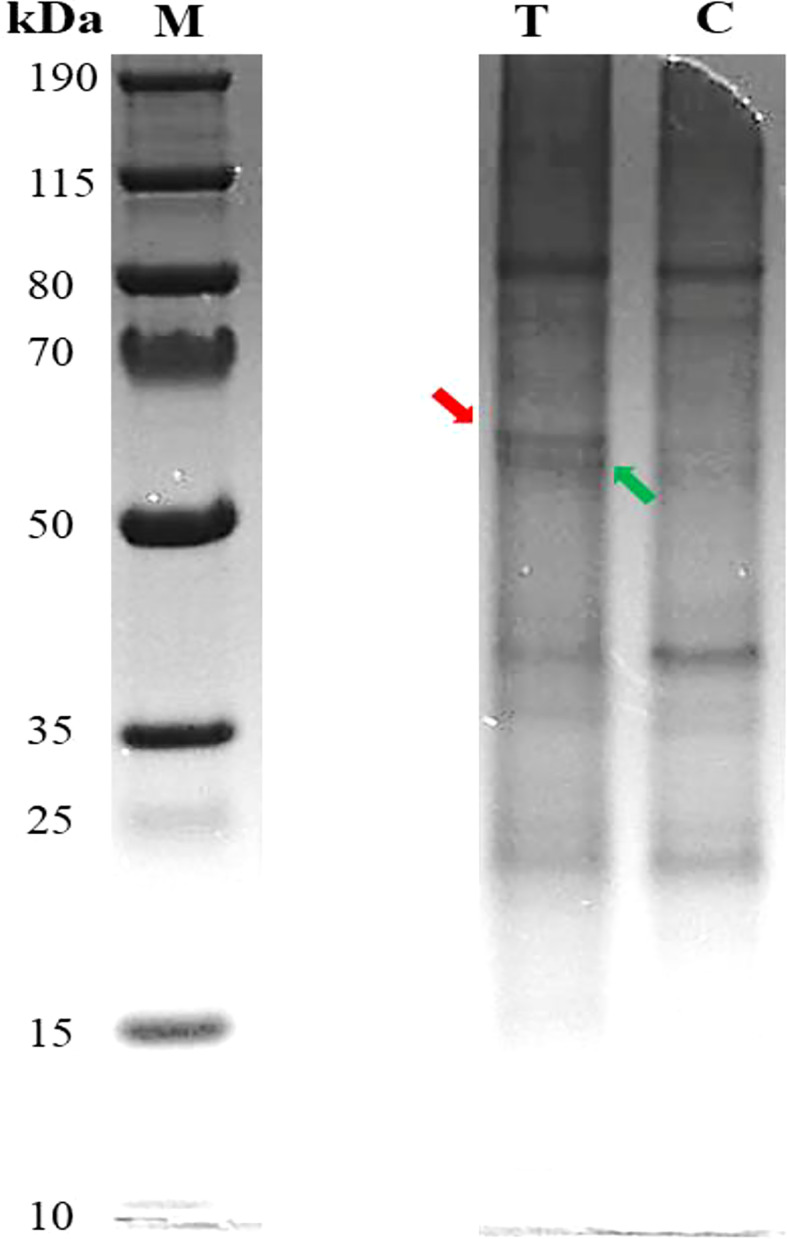
Pb-treated *T. qataranse* SDS-PAGE-separated proteins (whole plant). Lane 1, protein marker; lane 2, treatment (50 mg kg^⎯1^ Pb); and lane 3, control (0 mg kg^⎯1^ Pb). Approximately 20 µg of proteins was resolved using NuPAGE 4%–12% Bis-Tris Gels (Invitrogen) and visualized by Imperial™ Protein Stain. The figure shows that marker (M), treatment (T), and control (C) lanes were excised from the same original gel representing the loading concentration reported in this work.

**Figure 2:**
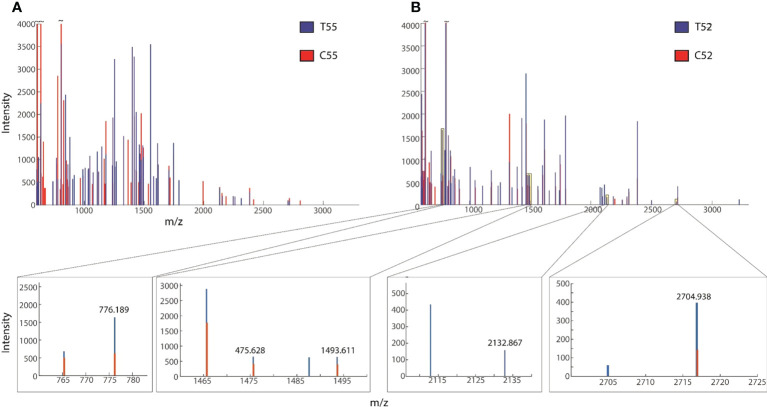
Overplayed MALDI-TOF/TOF mass lists of spectra obtained from **(A)** ~55-kDa MW and **(B)** ~52-kDa MW band areas in **Figure 1**. Blue and red peaks correspond to treatment (T52, T49) and control (C52, C49), respectively.

The results of Pb-treated *T. qataranse* (whole plant) separated proteins by SDS-PAGE are shown in **Figure 1**. Proteins were resolved using NuPAGE 4%–12% Bis-Tris Protein Gels (Invitrogen) and visualized by Imperial™ Protein Stain (Thermo Fischer Scientific). **Figure 1** shows that marker (M), treatment (T), and control (C) lanes were excised from the same original gel representing the loading concentration reported in this work. The green arrow indicates probable induction of catalase (CAT) (Romero‐Puertas et al., 2002), glutathione reductase (GR) (Romero‐Puertas et al., 2006), or phytochelatin synthase (PCS) (Filiz et al., 2019) at approximately ~52 kDa while the red arrow shows prominent polypeptide Rubisco (large subunit), a characteristic feature of plant tissue protein extracts at ~55 kDa (Walliwalagedara et al., 2010). **Figure 2** show the overplayed MALDI-TOF/TOF mass lists of spectra obtained from ~55-kDa MW and ~52-kDa MW band areas in **Figure 1**.

### Gene ontology and protein enrichment analyses

**Figure 3:**
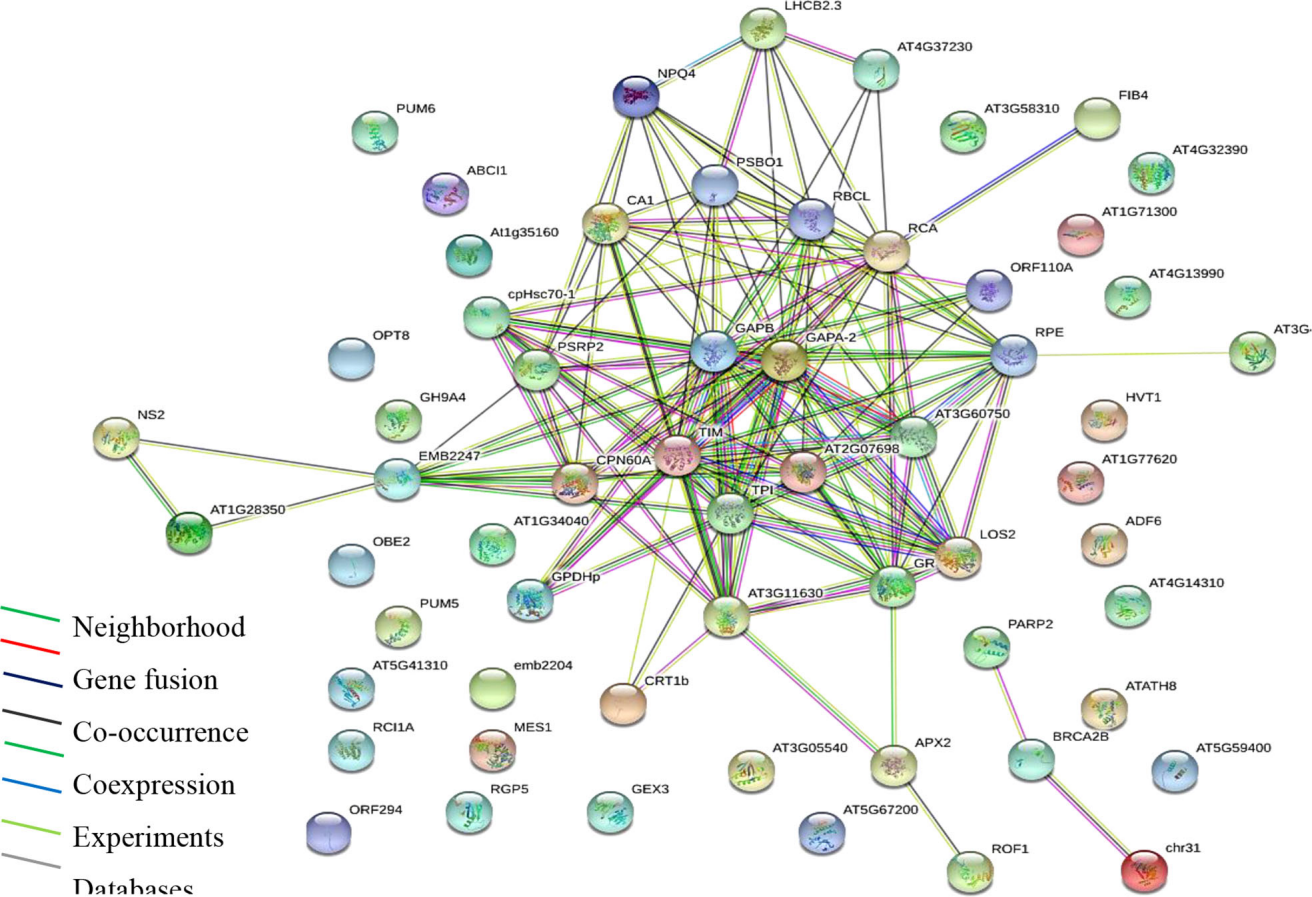
Protein interaction network (PPI) of identified proteins. Analysis performed using STRING *via* the https://string-db.org/cgi/network. PPI enrichment is significant (P ≤ 0.01).

Gene ontology and enrichment platforms provide useful tools for the functional annotation of gene products. Gene products are categorized into groups to understand their roles in a living system. In the present study, enrichment analyses were performed using different bioinformatics tools. First, we show a protein interaction network using the STRING database *via* https://string-db.org/ (**Figure 3**). Functional annotation based on molecular function, cellular process, and cellular component was performed in the UniProt database at https://www.uniprot.org/. Protein interaction network or PPI enrichment reveals a significant interaction between proteins (P ≤ 0.01), indicating that the proteins have more interaction than expected for a random set of proteins (**Figure 3**). The overall enrichment analysis showed that proteins with binding function dominate, followed by catalytic, transporter, and antioxidant molecules. The binding functions are to cations and anions, indicating that identified proteins constitute probable Pb binding domains.

Oxidoreductases top in catalytic binding function, which is also majorly composed of glutathione disulfide reductase and peroxides, suggesting the roles of antioxidative enzymes. Further, pathway enrichment analysis obtained from model plant pathway database “Plant Reactome” *via* https://plantreactome.gramene.org/ (**Table 1**) shows the enrichment of metabolic processes, regulation of HSF1-mediated heat shock response, cellular responses to stress, HSF1 activation, and glutathione metabolism, all of which have documented evidence in regulating tolerance to heavy metal stress in plants (Kumar and Prasad, 2018).

**Table 1:** Enriched pathways based on the Reactome Plant Pathways Database.

Term ID	Term description	Gene count	False discovery rate	Matching proteins in your network (IDs)
ATH-70171	Glycolysis	3	0.0049	AT2G21170.1, AT2G36530.1, AT3G55440.1
ATH-70263	Gluconeogenesis	3	0.0049	AT2G21170.1, AT2G36530.1, AT3G55440.1
ATH-71387	Metabolism of carbohydrates	4	0.0049	AT2G21170.1, AT2G36530.1, AT3G55440.1, AT3G60750.1
ATH-1445148	Translocation of SLC2A4 (GLUT4) to the plasma membrane	2	0.005	AT1G35160.2, AT5G38480.1
ATH-3371511	HSF1 activation	2	0.0183	AT1G35160.2, AT5G38480.1
ATH-2262752	Cellular responses to stress	3	0.048	AT1G35160.2, AT3G54660.1, AT5G38480.1
ATH-3371453	Regulation of HSF1-mediated heat shock response	2	0.048	AT1G35160.2, AT5G38480.1
ATH-00480	Glutathione metabolism	2	0.0467	AT3G09640.1, AT3G54660.1

### The identified proteins

Before matrix-assisted laser desorption/ionization time-of-flight mass spectrometry (MALDI-TOF MS) analysis, the gel containing separated proteins was image analyzed to determine the band intensity and sizes of the proteins using GelAnalyzer 2010a software (Bourven et al., 2012) and later tryptic digested. MALDI TOF/MS analysis was performed following Kwiecińska et al. (2018). Before bioinformatics analysis for protein identification, the preliminary inspection was carried out for differential mass lists. It was also reported that the raw peptide mass lists obtained higher and differential spectral peaks between treatment and the control. An example is shown in **Figure 3**, suggesting obvious differences in protein expression. Following MALDI-TOF/MS analysis, mass lists were searched in Mascot for peptide matches and protein identification in NCBI and UniProt databases. Only proteins with significant scores (P ≤ 0.05) are reported.

A list of the identified proteins with a wide spectrum of functions is shown in **Table 2**. Of these, six (6) proteins (AXX17_AT2G26660, AXX17_AT4G36160, AXX17_AT2G13500, AXX17_AT5G33340, AXX17_AT5G16980, and AXX17_AT1G74880) were of unknown function. Overall, the majority of the identified proteins showed that a large number of molecules were involved in metabolism and response to stress, including heavy metals (**Table 2**). Heat shock proteins, 70, 80–3, and 90 kDa, which are chloroplastic and function in both protein and ATP binding, and respond to abiotic stress (Ford et al., 2011, Majoul et al., 2003) were identified. Other ATPase-transporting chloroplastic proteins have also repeatedly appeared in varying molecular weights (MWs) and isoelectric points (pIs), such as Rubisco and chaperonin.

**Table 2:** Identified proteins and their gene ontology.

Accession	MW (Da)	pI	Homology	Protein name	GO
17367307	16,707.8	5.46	Arath	Actin-depolymerizing factor 6	B: Regulation of actin depolymerizing activity
297316109	70,755.3	5.58	Arath	Adenosylhomocysteinase	B: Carbon metabolism M: Adenosylhomocysteinase activity.
20140328	71,364.2	9	Arath	Asparagine-tRNA ligase	B: Protein biosynthesis C: Cytoplasm M: Aminoacyl-tRNA synthase
18404975	91,170.2	9.38	Arath	ATH subfamily protein ATH8	B: Regulation of ribosome binding C: Cytoplasm M: Ester bond activity
14423416	63,809.5	6.21	Arath	ATP synthase subunit beta-3	B: ATP synthesis C: Chloroplast M: ATP binding
5881679	55,328.5	5.19	Arath	ATPase subunit	B: ATP hydrolysis C: Chloroplast M: Catalyzing transmembrane movement
15226092	85,933.8	5.44	Arath	ATPase F1 complex alpha subunit protein	B: ATP Hydrolysis C: Chloroplast M: Poly(U) RNA and zinc ion binding
297318710	43,408.6	4.45	Arath	Calreticulin 2	B: Metabolic process C: Mitochondrion M: Calcium ion binding
3249100	50,581.9	5.46	Arath	Carbonic anhydrase	B: Carbon utilization C: Chloroplast M: Zinc ion binding
38503395	37,450.1	5.74	Arath	Carbonic anhydrase	B: Carbon utilization C: Chloroplast M: Metal ion binding
21554572	62,130.4	5.04	Arath	Chaperonin-60 alpha	B: Cellular protein metabolic process C: Chloroplast. M: ATP binding
115385	27,733.7	6.22	Araly	Chlorophyll a-b binding protein 4	B: Photosynthesis C: Chloroplast
116831194	28,063.2	6.53	Araly	Chlorophyll a/b-binding protein	B: Photosynthesis C: Chloroplast M: Chlorophyll and metal ion binding.
30697525	30,008.9	8.24	Arath	D-Ribulose-5-phosphate-3-epimerase	B: Carbohydrate metabolic process C: Chloroplast M: Ribulose-phosphate 3-epimerase activity
15225693	25,921.5	5.25	Arath	Dienelactone hydrolase domain-containing protein	B: Nucleoside metabolic process C: Chloroplast M: Hydrolase and transferase activity
75161476	54,120.1	9.07	Arath	Endoglucanase 16	B: Carbohydrate metabolism C: Mitochondrion M: Protein and single-stranded DNA binding
7433553	59,304.7	5.89	Araly	Enolase	B: Glycolysis M: Phosphopyruvate hydratase activity
10086473	51,654.2	6.13	Araly	Flavin-containing monooxygenase family protein	B: Auxin biosynthesis M: Flavin adenine dinucleotide binding
297326214	43,059.2	6.27	Araly	Fructose biphosphate adolase	B: Glucose catabolic process C: Cytoplast M: Fructose-biphosphate adolase activity
297338561	42,703.9	7.62	Araly	GAPA	B: Protein modification C: Chloroplast M: NAD, NADP, and nucleotide binding
8778823	10,7175.8	9.1	Arath	GTP binding Elongation factor Tu family protein	B: Response to cadmium ion C: Chloroplast M: GTP binding
15222111	42,847	8.16	Arath	Glyceraldehyde 3-phosphate dehydrogenase (GAPA2)	B: Glycolysis C: Chloroplast M: NAD and NADP binding
297326214	43,059.2	6.18	Araly	Glyceraldehyde-3-phosphate dehydrogenase	B: Protein modification M: Oxidoreductase activity
9758815	40,548.1	8.79	Arath	Glycolate oxidase	B: Defense response to bacterium C: Chloroplast M: Catalytic and oxidoreductase activity
591401946	60,991.4	5.66	Arath	Glycosyltransferase	B: Glycosylation
297310259	78,479.1	5.01	Araly	Heat shock protein 81-3	B: Stress response C: Nucleolus M: ATP binding
297318892	71,428.2	5.06	Araly	Heat shock protein 70	B: Response to cadmium ion C: Cytoplasm. M: ATP binding
219766617	77,106	5.13	Arath	Heat shock protein 70	B: Response to cadmium ion C: Chloroplast M: ATP binding
1032296797	7,540	9.52	Arath	Protein AXX17_AT2G26660	Unknown
1032282636	304,279	4.72	Arath	Protein AXX17_AT4G36160	Unknown
OAP09027	61,847	7.53	Arath	Protein AXX17_AT2G13500	Unknown
1032280307	49,017	5.77	Arath	Protein AXX17_AT5G33340	Unknown
1032277271	66,677	8.77	Arath	Protein AXX17_AT5G16980	Unknown
OAP18313	35,666	5.74	Arath	Protein AXX17_AT1G74880	Unknown
297328438	43,331.5	8.63	Araly	Kinase family protein	B: Chloroplast relocation C: Chloroplast M: ATP binding
15237622	108,115.3	8.77	Arath	Kinesin-like protein	B: DNA methylation M: Nucleic acid-binding
297322140	27,214.2	5.88	Arath	L-ascorbate peroxidase 2	B: Stress response C: Chloroplast M: Metal ion binding
118572828	28,006.2	5.88	Arath	L-ascorbate peroxidase 2	B: Stress response M: Heme binding and peroxidase activity
297334190	52,966.4	5.96	Araly	Large subunit of Rubisco	B: Protein modification C: Chloroplast M: Magnesium ion binding
297332730	52,973.4	6	Araly	Large subunit of ribulose-1,5-bisphosphate carboxylase/oxygenase	B: Carbamylation of the active site M: Magnesium ion binding
15239602	28706.8	4.96	Arath	Light-harvesting chlorophyll B-binding protein 3	B: Photosynthesis C: Membrane
75330960	29,766.5	4.97	Arath	Methyl-esterase 1	B: Fatty acid catabolic process C: Extracellular region
15221728	25,845	9.82	Arath	Non-intrinsic ABC protein 10_AT1G63270	B: C: M:
67460972	82,079.5	9.06	Arath	Oligopeptide transporter 8	B: Oligopeptide transport C: Nucleus M:NAD+ ADP-ribosyltransferase activity
19883896	53,397.9	5.55	Arath	Oxygen-evolving enhancer protein 1-1	B: Photosynthesis C: Chloroplast
12323399	128,658.4	4.97	Arath	P-loop containing nucleoside triphosphate hydrolases superfamily protein	B: Protein modification C: Nucleus M: Nucleotide-binding
297330766	50,007.9	6.08	Araly	Phosphoglycerate kinase	B: Glycolysis M: Phosphoglycerate kinase activity.
297312819	35,189.2	6.1	Araly	Photosystem II oxygen-evolving complex protein 1	B: Photosynthesis C: Chloroplast M: Calcium ion binding
15232249	28,802.9	5.62	Araly	Photosystem II light-harvesting complex protein 2.3	B: Cellular response to water deprivation C: Golgi apparatus M: Chlorophyll binding
297334193	39,547.8	5.46	Araly	Photosystem II protein D2	B: Electron transporter C: Chloroplast
297337134	28,054	9.24	Araly	Photosystem II subunit S	B: Cysteine biosynthesis C: Plastid thylakoid
15235490	23,051.8	9.85	Arath	Photosystem I subunit L	B: Cellular cation homeostasis C: Chloroplast
297332701	82,475.7	6.89	Ara	Photosystem I P700 chlorophyll a Apo protein A2	B: Chlorophyll biosynthesis C: Chloroplast M: Chlorophyll binding
297334183	56,053.4	6.4	Araly	Photosystem II 47kDa protein	B: Electron transport C: Chloroplast M: Chlorophyll binding
15235478	15,686.3	5.01	Arath	Photosystem II manganese-stabilizing protein (PsbO)	B: Photosynthesis C: Chloroplast M: Calcium ion binding
17380270	28,007.9	9.25	Arath	Photosystem II 22-kDa protein	B: Photosystem II stabilization C: Chloroplast M: Xanthophyll binding
75163506	74,006.6	8.17	Arath	Probable inactive receptor kinase At5g67200	B: Protein phosphorylation C: Plasma membrane ATP binding and protein kinase activity
75170207	38,585.6	5.07	Arath	Probable UDP-arabinopyranose mutase 5	B: Response to salt stress M: Nucleic acid and zinc ion binding
1709740	72,176	5.92	Arath	Poly[ADP-ribose] polymerase 2	B: Protein ADP-ribosylation C: Chloroplast
42570340	144,479.7	8.25	Arath	Protein helicase in vascular tissue and tapetum	B: Cytokinesis M: ATP binding
334187718	110,499	5.64	Arath	Protein embryo defective 2247_AT5G16715	B: C: M:
42572779	40,737.6	9.44	Arath	Protein FORKED 1	C: Cytoplasm M: Actin binding
297334210	83,199.1	6.6	Araly	psi P700 Apo protein A1	B: Electron transport C: Thylakoid
297332727	51,868.1	6.7	Araly	PS II 43-kDa protein	B: Electron transporter C: Membrane M: Chlorophyll binding
313471415	106,995.7	5.61	Arath	Pumilio homolog 5	B: Translation regulation C: Cytoplasm M: RNA binding
75168940	96,105.5	6.96	Arath	Pumilio homolog 6	B: Translation regulation C: Chloroplast
75182934	29,474.5	9.2	Arath	Putative cysteine-rich repeat secretory protein 61	C: Chloroplast M: mRNA binding
42558968	12,664.6	9.14	Arath	Putative uncharacterized mitochondrial protein AtMg00280	B: Carbon fixation C: Chloroplast M: ATP binding
3914541	52,955.3	5.88	Arath	Ribulose biphosphate carboxylase large chain	B: Carbon fixation C: Membrane
15229244	52,434.6	5.53	Arath/Araly	RING/FYVE/PHD zinc finger-containing protein	B: Transferring phosphorus-containing groups M: DNA binding
79314769	27,748.6	8.94	Arath	RNA recognition motif-containing protein	B: RNA processing C: Chloroplast M: RNA binding
297337086	47,782.9	8.99	Arath	Serine hydroxymethyltransferase	B: L-serine metabolic process C: Mitochondrion M: Pyridoxal phosphate binding
544602156	158,724.2	5.76	Arath	SNF2 domain-containing protein CLASSY 3	M: ATP binding
79587640	100,474.8	7.25	Arath	Transducin/WD40 domain-containing protein-like protein	C: Cell wall M: Protein binding
7329685	81,475.4	5.8	Arath	Transketolase	B: Acetyl-coA metabolic process C: Chloroplast M: Transketolase activity
297322418	79,851.5	5.85	Araly	Transketolase	B: Acetyl-coA metabolic process C: Chloroplast M: Metal ion binding and transketolase activity
8778823	107,175.8	9.1	Arath	Translation elongation factor eEF-1 alpha chain	B: Protein biosynthesis M: Translation elongation factor activity
13431953	33,345.9	5.39	Araly	Triosephosphate isomerase	B: Golgi organization C: Chloroplast M: Catalytic activity
13432260	27,169.2	5.39	Arath	Triosephosphate isomerase	B: Golgi organization C: Cell wall M: Copper ion binding
75170045	51,654.2	8.22	Arath	Tryptophan aminotransferase-related protein 3	B: Auxin biosynthesis C: Extracellular region M: Catalytic and pyridoxal phosphate binding
297335760	53,397.9	6.45	Arath	Tyrosyl-tRNA synthetase-like	B: Chloroplast organization C: Chloroplast M: ATP binding
75172681	80,713.7	5.38	Arath	Vacuolar protein sorting-associated protein 52 B	C: Chloroplast M: NAD binding
332641995	40,934.4	6.91	Arath	2-Cys peroxiredoxin (2-Cys PrxA)	B: Cuticle development C: Chloroplast
14916972	29,092.2	6.91	Arath	2-Cys peroxiredoxin BAS1	C: Chloroplast M: Protein binding
1702987	30,194.1	4.79	Arath	14–3–3-like protein GF14 phi	B: Response to cadmium ion C: Cytoplasm M: FK506 binding
110740990	36,144.5	5.55	Arath	33-kDa polypeptide of oxygen-evolving complex	B: Photosynthesis C: Chloroplast
73919362	61,453.2	5.24	Arath	70-kDa peptidyl-prolyl isomerase ROF1	C: Chloroplast M: Phosphatidylinositol binding

Accession number was obtained from NCBI based on search against the Arabidopsis database. MW, molecular weight; pI, isoelectric point; GO, Gene Ontology; Araly, Arabidopsis lyrata; Arath, Arabidopsis thaliana; B, biological process; C, cellular component; M, molecular function.

Interestingly, some proteins such as transketolase, flavin-containing monooxygenase family protein, pumilio homolog 6 and 5, and 14–3–3-like protein GF14 psi were also identified. Others such as carbonic anhydrase and the flavin-containing monooxygenase family respond to cadmium stress and play important roles in auxin biosynthesis. Carbonic anhydrase is involved in glycophyte-assisted phytoremediation of Zn, Pb, and Cd (Tangahu et al., 2011). While Zhao et al. (2001) noted that the flavin-containing monooxygenase family protein, which regulates auxin biosynthesis, also mediates the translocation of these metals across plant tissues, it was also shown to play a vital role in the xenobiotic detoxification mechanism by directing the correct folding of protein-containing sulfide bonds (Naumann et al., 2002); it may have a similar role in Pb detoxification in *T. qataranse.* Further, the two pumilio homolog proteins were both chloroplastic and cytosolic with binding functions that were also suggested to be emerging regulators for plants’ response to environmental constraints (Ambrosone et al., 2012).

## Discussion

In this study, protein identification and gene enrichment analysis reveal several differentially expressed molecules due to Pb stress. Increased protein synthesis due to Pb stress in plants is one of the major cellular metabolic processes (Kohli et al., 2018). For instance, the mitogen-activated protein (MAP) kinase pathways regulate such processes, which serve as a signaling system against oxidative stress (Mapanda et al., 2005). Such signaling occurs through multiple stages of the reaction, which modify gene expression and ultimately protein synthesis (Sidhu et al., 2016). Therefore, studying the differential expression pattern of such proteins provides insight into the mechanism of plant-metal interaction, which helps develop transgenic species of plants with enhanced metal tolerance and a detoxification system for phytoremediation. Recently, Wang et al. (2015) identified 16,246 uniquely expressed genes in *Platanus acerifolia* due to Pb exposure. Of the differentially identified unigenes, antioxidant proteins, metal chelators, and transporters dominate, while glutathione and other metabolic pathways were found to play role in the defense and detoxification of Pb.

In this work, key stress-regulated metabolic pathways including glutathione metabolism, cellular response to stress, and HSF1-mediated heat shock response regulation were identified. The heat shock proteins (HSPs) are one of the most abundant of the stress-responsive proteins, suggesting its crucial role in Pb detoxification. Indeed, HSP induction has proven to play a critical protective role, confer organisms with eco-physiological adaptation, and genetically conserved response to environmental stress. A similar study involving Pb-exposed *Acalypha indica* Venkatachalam et al. (2017) found differentially expressed proteins to contain heat shock proteins (HSP). These functions in plants defense against oxidative stress and maintain cellular homeostasis (Kumar and Majeti, 2014). Additionally, HSP is involved in translocation, degradation and prevents protein aggregation during transport in stressed environments (Wang et al., 2011).

Furthermore, in a comparative proteomic study of Pb stress in a related halophyte *S. salsa*, Liu et al. (2016) reported significant differential expressions of proteins. The majority of the identified proteins were involved in defense-related metabolic pathways. Some of the proteins include carbonic anhydrase, ribulose 1,5 bisphosphate, chlorophyll a*-b* binding protein, and glutathione peroxidase, all of which were also identified in the present study.

In a critical review of Pb-induced stress in plants, Kumar and Prasad (2018) showed the critical roles of ROS in the metal tolerance, uptake, and detoxification mechanism. In addition to binding and stress response proteins, enrichment analysis showed major oxidoreductases. Heavy metals are considered a primary source of injury to the cell membrane, frequently attributed to lipid peroxidation. Excessive ROS production causes oxidative stress, as reported for many crops under heavy metals treatment, and is likely to be commenced by molecular oxygen excitation (O_2_) to generate singlet oxygen or by electron transfer to O_2_ and genesis of free radicals, i.e., O_2_^−^ and OH^−^ (Shakoor et al., 2014; Ma et al., 2022a; Ma et al., 2022b; Ma et al., 2022c). Our group noted increased activities of key antioxidant enzymes, superoxide dismutase, catalase, glutathione reductase, and peroxidases in Pb treated *T. qataranse*. In this work, the enriched glutathione catalytic enzymes and metabolic pathway suggest PC induction due to Pb stress. Enzymes involved in glutathione metabolism mediate metal detoxification (Anjum et al., 2012). Glutathione-S-transferases (GSTs) are primary phase II GSH-dependent ROS scavenging enzymes. They play essential roles in GSH conjugation with exogenous and endogenous species found during oxidative stress, including H_2_O_2_ and lipid peroxides (Kumar and Majeti, 2014). Glutathione metabolism regulates the biosynthesis of phytochelatins (PC), which bind Pb and transports it to vacuoles where detoxification can occur. GSH and phytochelatin (PCS)-related genes are actively involved in GSH-dependent PC synthesis (Fan et al., 2016).

Meanwhile, all the six hypothetical proteins AXX17_AT2G26660, AXX17_AT4G36160, AXX17_AT2G13500, AXX17_AT5G33340, AXX17_AT5G16980, and AXX17_AT1G74880 with unknown function are probably involved in Pb chelation and or transport. These proteins were obtained from ~55- and ~ 52-kDa MW areas (**Figure 1**). Inspection of the protein sequences showed that protein AXX17_AT2G26660 is rich in glycine and composed of up to 76% residues, suggesting that it belongs to the glycine-rich proteins (GRPs) and may therefore have some role in Pb tolerance in *T. qataranse*. GRPs are characterized by high glycine content and the presence of conserved segments, including glycine-containing structural motifs. They are involved in the cellular response to stress (biotic and abiotic), including salinity, temperature, and drought in plants (Ortega-Amaro et al., 2015). GRPs’ functional diversity and their roles in response to stress in plants are well documented in separate reviews by Mangeon et al. (2010) and Czolpinska and Rurek (2018), respectively. In plants, prominent stress-responsive proteins such as metallothioneins and phytochelatins ameliorate metal toxicity, including Pb, through binding and aiding in vacuolar sequestration. However, to the best of our knowledge, there is no report on GRPs’ role in Pb tolerance, bioaccumulation, or detoxification in plants. In addition, the expression of the other hypothetical proteins due to Pb stress suggests their potential roles in Pb tolerance and detoxification in *T. qataranse.*

## Conclusion

A total of eighty-six (86) differentially expressed proteins, the majority of which function in ion and protein binding, antioxidant activity, transport, and abiotic response stress, were identified. In addition, essential stress-regulating metabolic pathways, including glutathione metabolism, cellular response to stress, and regulation of HSF1-mediated heat shock response, were also enriched. Indeed, HSP induction has proven to play a critical protective role, confer organisms with eco-physiological adaptation, and genetically conserve response to environmental stress. Further, enrichment analysis showed six (6) proteins with unknown functions. Additionally, at 52- and 49-kDa MW band areas, six (6) hypothetical proteins with unknown functions were identified. Of these, protein AXX17_AT2G26660 is highly rich in glycine amino acid residues (up to 76%), suggesting that it may belong to the “glycine-rich proteins (GRPs).” In plants, prominent stress-responsive proteins such as metallothioneins and phytochelatins ameliorate metal toxicity, including Pb, through binding and aiding in vacuolar sequestration. Although GRPs are known to be involved in plant defense against abiotic stress, including salinity, and drought, there is no report on their role on Pb tolerance and or detoxification in plants. Enrichment analysis in the current study reveals that the hypothetical proteins do not interact with known proteins and are not part of any enriched pathway. We conclude that the hypothetical proteins belong to the GRP superfamily, are potential novel Pb chelators, and may play an essential role in the Pb detoxification in *T. qataranse.* However, further functional studies are required to elucidate their specific functions.

## Materials and methods

### Total protein extraction

For proteomic analysis, 7-week-old *T. qataranse* plants treated with 50 mg kg^⎯1^ Pb were ground to a fine powder using liquid nitrogen and total protein extracted using phenol/SDS buffer as described by Wang et al. (2006). Approximately 330 mg of leaf/root tissues was ground to a fine powder with liquid nitrogen in a mortar and pestle. Powdered tissue collected in 2-ml microcentrifuge tubes and 1 ml of 10% (v/v) TCA/acetone were added, vortexed, and centrifuged at 4°C and 14,000 rpm for 3 min. Following the first centrifugation, the tissue supernatant was discarded, and 1 ml of 80% (v/v) methanol and 0.1 M ammonium acetate acetone were added, vortexed, and centrifuged for another 3 min at 4°C and 14,000 rpm. The supernatant from the second centrifugation was again discarded, and 1 ml of 80% (v/v) acetone was added and vortexed until the pellets were fully dispersed. Finally, it was centrifuged at 4°C and 14,000 rpm for 3 min and the supernatant discarded. The settled pellets at the bottom of the tubes were air-dried at room temperature to remove residual acetone. About 400 µl each for phenol and 10% (w/v) SDS buffer in 5% (v/v) of β-mercaptoethanol were added, thoroughly mixed, and centrifuged for 3 min at 14,000 rpm following 5 min of ice incubation. The upper phenol phase transferred to new 2-ml microcentrifuge tubes consisted of 1.2 ml of 0.1 M ammonium acetate added and incubated overnight at -20°C. The incubated mixture of the above was centrifuged at 4°C and 14,000 rpm for 5 min, and the supernatant was discarded. One milliliter of 100% methanol was added, vortexed, and centrifuged and the supernatant discarded. The extracted protein sample was air-dried at room temperature, resuspended in 200 µl sample buffer (2× Laemmli) with 5% (v/v) β-mercaptoethanol, and kept at -80°C.

### Determination of protein concentration by Bradford assay

According to Bio-Rad protocols, the assay was performed as described in Li et al. (2013) to measure the quantity of the protein extracted from the various extraction methods above in terms of concentration and yield. The assay was carried out using bovine serum albumin (BSA) as standard. Series of BSA concentrations 0, 1, 2, 4, 6, 8, and 10 µg ml^⎯1^ were prepared from the stock and topped up with different volumes of distilled water to a volume of 0.5 ml. At the same time, protein samples were diluted approximately 100× in the same volume containing 10 µl of protein samples and 490 µl of distilled water. The exact amount of 0.5 ml of Bradford reagent was added to both the standards and sample to a total volume of 1 ml and mixed by shaking the tubes. The mixture was incubated for 20 min and the Bradford reagent reaction triggered to both the protein standard and sample. The absorbance of the reactions were measured at 595 nm using a spectrophotometer (GENESYS UV-Vis spectrophotometer) against the blank. The measure of absorbance taken from the standard plotted against each BSA concentration was used to determine protein sample concentrations and yields using the equation of the standard curve.

### Sodium dodecyl sulfate-polyacrylamide gel electrophoresis (SDS-PAGE)

One-dimensional SDS-PAGE was carried out in this investigation to separate proteins based on size. Approximately 20 µg of extracted proteins was resolved using NuPAGE 4%–12% Bis-Tris Protein Gels (Invitrogen) and separated by SDS-PAGE according to Laemmli (1970) on a Bio-Rad Protean II system. Loaded samples were run for 15 min at 100 V and later for 70 min at 150 V. Subsequently, gels were stained with Coomassie Stain G-250 and destained overnight for further protein pattern analysis according to band intensity.

### Trypsin digestion

To prepare the extracted samples for MALDI-TOF MS and the eventual generation of mass protein data for protein identification, digestion was carried out with sequencing-grade trypsin (Shevchenko et al., 2000). The protein concentration of 50 µg was prepared in 100 µl total volume with 50 mM NH_4_HCO_3_. About 5 µl of 200 mM DTT (in 100 mM NH_4_HCO_3_) was then added to reduce the sample by boiling for 10 min followed by incubation for 50 min at room temperature. To alkylate the samples, 4 µl 1 M iodoacetamide was added, vortexed, briefly spun, and incubated for 50 min at room temperature. Iodoacetamide was neutralized by the addition of 20 µl 200 mM DTT, vortexed, spun, and incubated at room temperature for 50 min. Digestion was carried out in a ratio of 1:20 of trypsin to the sample. Samples were later vortexed and briefly spun prior to overnight incubation at 37°C. The pH was neutralized by adding 2% formic to the sample repeatedly and monitored until it reached 6.0 using pH paper indicators. Finally, digested protein samples were cleaned with C18 ZipTip and kept at -80°C for MALDI-TOF MS analysis.

### MALDI-TOF/MS and bioinformatics analysis

Proteins were eluted in 50% acetonitrile containing α-cyano-4-hydroxycinnamic acid directly applied onto the target metal plate and analyzed by MALDI-TOF MS on a Bruker Ultraflex (Bruker Daltonics, Bremen, Germany). Peptide mass spectra were analyzed using embedded flexAnalysis software. Calibration of peptide spectra was internally performed using trypsin autolytic proteins (842.51, 1,045.56, and 2,211.10 Da). Protein identity search was performed using the Mascot protein identification server *via* http://www.matrixscience.com/ using the model plant “*Arabidopsis*” protein database. The set parameters included one miscut, alkylation, and partial oxidation of methionine. Others were *Arabidopsis thaliana* such as taxonomy, pI, and Mr determined from gel migration spot position with mass tolerance of 0.1 Da. The statistical significance of all identified proteins was evaluated according to Z-value and sequence coverage based on Mascot algorithms. Subsequent gene ontology and enrichment analysis were performed using Gene Ontology Resource (http://geneontology.org/) and UniProt database *via* (https://www.uniprot.org/).

### Statistical analysis

All data were statistically analyzed using one-way ANOVA with the statistical package Sigma Plot, Systat Software Inc., and treatment means compared by Tukey test (Steel et al., 1997). Statistical significance was considered at *P* < 0.05.

## Data availability statement

The datasets presented in this study can be found in online repositories. The names of the repository/repositories and accession number(s) can be found in the article/supplementary material.

## Author contributions

KU and MHA-D designed the experiment. KU conducted the experiment. KU and SS ran MALDI TOF/MS analyzed and received the data sets. KU, SS, MAA-G, and NZ analyzed the data for protein identification. KU wrote the manuscript. SS, NZ, and MHA-D revised the manuscript. All authors contributed to the article and approved the submitted version.

## Funding

Qatar University’s student grant QUST-CAS-SPR-2017-33 supports this study.

## Acknowledgments

The authors wish to acknowledge Dr. Hanaa Mousa of the College of Medicine, Qatar University, for her support in MALDI TOF/MS analysis. Finally, we thank Dr. Chaevien S. Clendinen for the critical reading of this manuscript. Qatar National Library provides open access funding.

## Conflict of interest

The authors declare that the research was conducted in the absence of any commercial or financial relationships that could be construed as a potential conflict of interest.

## Publisher’s note

All claims expressed in this article are solely those of the authors and do not necessarily represent those of their affiliated organizations, or those of the publisher, the editors and the reviewers. Any product that may be evaluated in this article, or claim that may be made by its manufacturer, is not guaranteed or endorsed by the publisher.

## References

[B1] Agrawal G. K. Pedreschi R. Barkla B. J. Bindschedler L. V. Cramer R. Sarkar A.. (2012). Translational plant proteomics: A perspective. J. Proteomics 75, 4588–4601. doi: 10.1016/j.jprot.2012.03.055 22516432

[B2] Ahmad S. Mfarrej M. F. B. El-Esawi M. A. Waseem M. Alatawi A. Nafees M.. (2022). Chromium-resistant staphylococcus aureus alleviates chromium toxicity by developing synergistic relationships with zinc oxide nanoparticles in wheat. Ecotoxicology. Environ. Saf. 230, 113142. doi: 10.1016/j.ecoenv.2021.113142 34990991

[B3] Ali H. Khan E. Sajad M. A. (2013). Phytoremediation of heavy metals–concepts and applications. Chemosphere 91, 869–881. doi: 10.1016/j.chemosphere.2013.01.075 23466085

[B4] Alsafran M. Usman K. Ahmed B. Rizwan M. Saleem M. H. Al Jabri H. (2022). Understanding the phytoremediation mechanisms of potentially toxic elements: A proteomic overview of recent advances. Front. Plant Sci. 13. doi: 10.3389/fpls.2022.881242 PMC9134791 35646026

[B5] Ambrosone A. Costa A. Leone A. Grillo S. (2012). Beyond transcription: RNA-binding proteins as emerging regulators of plant response to environmental constraints. Plant Sci. 182, 12–18. doi: 10.1016/j.plantsci.2011.02.004 22118611

[B6] Anjum N. A. Ahmad I. Mohmood I. Pacheco M. Duarte A. C. Pereira E.. (2012). Modulation of glutathione and its related enzymes in plants’ responses to toxic metals and metalloids–A review. Environ. Exp. Bot. 75, 307–324. doi: 10.1016/j.envexpbot.2011.07.002

[B7] Baerenfaller K. Grossmann J. Grobei M. A. Hull R. Hirsch-Hoffmann M. Yalovsky S.. (2008). Genome-scale proteomics reveals arabidopsis thaliana gene models and proteome dynamics. Science 320, 938–941. doi: 10.1126/science.1157956 18436743

[B8] Bhantana P. Rana M. S. Sun X.-c. Moussa M. G. Saleem M. H. Syaifudin M.. (2021). Arbuscular mycorrhizal fungi and its major role in plant growth, zinc nutrition, phosphorous regulation and phytoremediation. Symbiosis 84 (1), 19–37. doi: 10.1007/s13199-021-00756-6

[B9] Bourven I. Costa G. Guibaud G. (2012). Qualitative characterization of the protein fraction of exopolymeric substances (EPS) extracted with EDTA from sludge. Bioresour. Technol. 104, 486–496. doi: 10.1016/j.biortech.2011.11.033 22154750

[B10] Cobbett C. S. (2000). Phytochelatin biosynthesis and function in heavy-metal detoxification. Curr. Opin. Plant Biol. 3, 211–216. doi: 10.1016/S1369-5266(00)00066-2 10837262

[B11] Czolpinska M. Rurek M. (2018). Plant glycine-rich proteins in stress response: An emerging, still prospective story. Front. Plant Sci. 9, 302. doi: 10.3389/fpls.2018.00302 29568308 PMC5852109

[B12] Fan T. Yang L. Wu X. Ni J. Jiang H. Zhang Q. A.. (2016). The PSE1 gene modulates lead tolerance in Arabidopsis. J. Exp. Bot. 67 (15), 4685–4695. 27335453 10.1093/jxb/erw251 PMC4973742

[B13] Filiz E. Saracoglu I. A. Ozyigit I. I. Yalcin B. (2019). Comparative analyses of phytochelatin synthase (PCS) genes in higher plants. Biotechno. Biotechnolog. Equipment 33 (1), 178–194.

[B14] Ford K. L. Cassin A. Bacic A. (2011). Quantitative proteomic analysis of wheat cultivars with differing drought stress tolerance. Front. Plant Sci. 2, 44. 22639595 10.3389/fpls.2011.00044 PMC3355674

[B15] Fulekar M. Singh A. Bhaduri A. M. (2009). Genetic engineering strategies for enhancing phytoremediation of heavy metals. Afr. J. Biotechnol. 8 (4).

[B16] Ghani M. A. Abbas M. M. Ali B. Aziz R. Qadri R. W. K. Noor A.. (2021). Alleviating role of gibberellic acid in enhancing plant growth and stimulating phenolic compounds in carrot (Daucus carota l.) under lead stress. Sustainability 13, 12329. doi: 10.3390/su132112329

[B17] Haas W. Faherty B. K. Gerber S. A. Elias J. E. Beausoleil S. A. Bakalarski C. E.. (2006). Optimization and use of peptide mass measurement accuracy in shotgun proteomics. Mol. Cell. Proteomics 5, 1326–1337. doi: 10.1074/mcp.M500339-MCP200 16635985

[B18] Jabeen R. Ahmad A. Iqbal M. (2009). Phytoremediation of heavy metals: Physiological and molecular mechanisms. Botanical. Rev. 75, 339–364. doi: 10.1007/s12229-009-9036-x

[B19] Jiang M. Liu S. Li Y. Li X. Luo Z. Song H.. (2019). EDTA-facilitated toxic tolerance, absorption and translocation and phytoremediation of lead by dwarf bamboos. Ecotoxicol. Environ. Saf. 170, 502–512. doi: 10.1016/j.ecoenv.2018.12.020 30557708

[B20] Kanwal U. Ali S. Shakoor M. B. Farid M. Hussain S. Yasmeen T.. (2014). EDTA ameliorates phytoextraction of lead and plant growth by reducing morphological and biochemical injuries in brassica napus l. under lead stress. Environ. Sci. pollut. Res. 21, 9899–9910. doi: 10.1007/s11356-014-3001-x 24854501

[B21] Kärenlampi S. Schat H. Vangronsveld J. Verkleij J. van der Lelie D. Mergeay M.. (2000). Genetic engineering in the improvement of plants for phytoremediation of metal polluted soils. Environ. Pollut. 107, 225–231. doi: 10.1016/S0269-7491(99)00141-4 15092999

[B22] Kohli S. K. Handa N. Bali S. Arora S. Sharma A. Kaur R.. (2018). Modulation of antioxidative defense expression and osmolyte content by co-application of 24-epibrassinolide and salicylic acid in Pb exposed Indian mustard plants. Ecotoxicol. Environ. Saf. 147, 382–393. doi: 10.1016/j.ecoenv.2017.08.051 28881317

[B23] Kosová K. Vítámvás P. Prášil I. T. Renaut J. (2011). Plant proteome changes under abiotic stress–contribution of proteomics studies to understanding plant stress response. J. Proteomics 74, 1301–1322. doi: 10.1016/j.jprot.2011.02.006 21329772

[B24] Kumar A. Majeti N. V. P. (2014). Proteomic responses to lead-induced oxidative stress in talinum triangulare Jacq.(Willd.) roots: Identification of key biomarkers related to glutathione metabolisms. Environ. Sci. Pollut. Res. 21, 8750–8764. doi: 10.1007/s11356-014-2808-9 24705950

[B25] Kumar A. Prasad M. N. V. (2018). Plant-lead interactions: Transport, toxicity, tolerance, and detoxification mechanisms. Ecotoxicol. Environ. Saf. 166, 401–418. doi: 10.1016/j.ecoenv.2018.09.113 30290327

[B26] Kwiecińska A. Porwit A. Souchelnytskyi N. Kaufeldt A. Larsson C. Bajalica-Lagercrantz S.. (2018). Proteomic profiling of diffuse Large b-cell lymphomas. Pathobiology 85 (4), 211–219. doi: 10.1159/000486285 29617697

[B27] Laemmli U. K. (1970). SDS-page Laemmli method. Nature 227, 680–685. 5432063 10.1038/227680a0

[B28] Li G. Peng X. Xuan H. Wei L. Yang Y. Guo T.. (2013). Proteomic analysis of leaves and roots of common wheat (Triticum aestivum l.) under copper-stress conditions. J. Proteome Res. 12, 4846–4861. doi: 10.1021/pr4008283 24074260

[B29] Liu X. Shen X. Lai Y. Ji K. Sun H. Wang Y.. (2016). Toxicological proteomic responses of halophyte suaeda salsa to lead and zinc. Ecotoxicol. Environ. Saf. 134, 163–171. doi: 10.1016/j.ecoenv.2016.07.017 27616546

[B30] Majoul T. Bancel E. Triboï E. Ben Hamida J. Branlard G. (2003). Proteomic analysis of the effect of heat stress on hexaploid wheat grain: Characterization of heat‐responsive proteins from total endosperm. Proteomics 3 (2), 175–183. 12601810 10.1002/pmic.200390026

[B31] Mangeon A. Junqueira R. M. Sachetto-Martins G. (2010). Functional diversity of the plant glycine-rich proteins superfamily. Plant Signal. Behav. 5, 99–104. doi: 10.4161/psb.5.2.10336 20009520 PMC2884108

[B32] Mapanda F. Mangwayana E. Nyamangara J. Giller K. (2005). The effect of long-term irrigation using wastewater on heavy metal contents of soils under vegetables in Harare, Zimbabwe. Agric. Ecosyst. Environ. 107, 151–165. doi: 10.1016/j.agee.2004.11.005

[B33] Ma J. Saleem M. H. Ali B. Rasheed R. Ashraf M. A. Aziz H.. (2022a). Impact of foliar application of syringic acid on tomato (Solanum lycopersicum l.) under heavy metal stress-insights into nutrient uptake, redox homeostasis, oxidative stress, and antioxidant defense. Front. Plant Sci. 13. doi: 10.3389/fpls.2022.950120 PMC9453224 36092395

[B34] Ma J. Saleem M. H. Yasin G. Mumtaz S. Qureshi F. F. Ali B.. (2022b). Individual and combinatorial effects of SNP and NaHS on morpho-physio-biochemical attributes and phytoextraction of chromium through cr-stressed spinach (Spinacia oleracea l.). Front. Plant Sci. 13. doi: 10.3389/fpls.2022.973740 PMC9428630 36061765

[B35] Ma J. ur Rehman M. Z. Saleem M. H. Adrees M. Rizwan M. Javed A.. (2022c). Effect of phosphorus sources on growth and cadmium accumulation in wheat under different soil moisture levels. Environ. Pollut. 311, 119977. doi: 10.1016/j.envpol.2022.119977 35987285

[B36] Nagajyoti P. C. Lee K. D. Sreekanth T. (2010). Heavy metals, occurrence and toxicity for plants: A review. Environ. Chem. Lett. 8, 199–216. doi: 10.1007/s10311-010-0297-8

[B37] Naumann C. Hartmann T. Ober D. (2002). Evolutionary recruitment of a flavin-dependent monooxygenase for the detoxification of host plant-acquired pyrrolizidine alkaloids in the alkaloid-defended arctiid moth tyria jacobaeae. Proc. Natl. Acad. Sci. 99, 6085–6090. doi: 10.1073/pnas.082674499 11972041 PMC122906

[B38] Ortega-Amaro M. A. Rodríguez-Hernández A. A. Rodríguez-Kessler M. Hernández-Lucero E. Rosales-Mendoza S. Ibáñez-Salazar A.. (2015). Overexpression of AtGRDP2, a novel glycine-rich domain protein, accelerates plant growth and improves stress tolerance. Front. Plant Sci. 5, 782. doi: 10.3389/fpls.2014.00782 25653657 PMC4299439

[B39] Parveen A. Saleem M. H. Kamran M. Haider M. Z. Chen J.-T. Malik Z.. (2020). Effect of citric acid on growth, ecophysiology, chloroplast ultrastructure, and phytoremediation potential of jute (Corchorus capsularis l.) seedlings exposed to copper stress. Biomolecules 10, 592. doi: 10.3390/biom10040592 PMC7226093 32290389

[B40] Pusztai A. (1966). The properties of bovine serum albumin and chymotrypsinogen A in solvent mixtures containing phenol. Biochem. J. 101 (2), 265. 5966264 10.1042/bj1010265 PMC1270105

[B41] Romero‐Puertas M. C. Palma J. M. Gómez M. Del Rio L. A. Sandalio L. M. (2002). Cadmium causes the oxidative modification of proteins in pea plants. Plant Cell Environ. 25 (5), 677–686.

[B42] Romero‐Puertas M. C. Corpas F. J. Sandalio L. M. Leterrier M. Rodríguez‐Serrano M. Del Río L. A.. (2006). Glutathione reductase from pea leaves: Response to abiotic stress and characterization of the peroxisomal isozyme. New Phytol 170 (1), 43–52. 16539602 10.1111/j.1469-8137.2006.01643.x

[B43] Saleem M. H. Ali S. Rehman M. Hasanuzzaman M. Rizwan M. Irshad S.. (2020a). Jute: A potential candidate for phytoremediation of metals–A review. Plants 9, 258. doi: 10.3390/plants9020258 PMC7076354 32079368

[B44] Saleem M. H. Fahad S. Khan S. U. Ahmar S. Khan M. H. U. Rehman M.. (2020b). Morpho-physiological traits, gaseous exchange attributes, and phytoremediation potential of jute (Corchorus capsularis l.) grown in different concentrations of copper-contaminated soil. Ecotoxicology. Environ. Saf. 189, 109915. doi: 10.1016/j.ecoenv.2019.109915 31722799

[B45] Saleem M. H. Fahad S. Khan S. U. Din M. Ullah A. Sabagh A. E. L.. (2020c). Copper-induced oxidative stress, initiation of antioxidants and phytoremediation potential of flax (Linum usitatissimum l.) seedlings grown under the mixing of two different soils of China. Environ. Sci. Pollut. Res. 27, 5211–5221. doi: 10.1007/s11356-019-07264-7 31848948

[B46] Saleem M. H. Parveen A. Khan S. U. Hussain I. Wang X. Alshaya H.. (2022). Silicon fertigation regimes attenuates cadmium toxicity and phytoremediation potential in two maize (Zea mays l.) cultivars by minimizing its uptake and oxidative stress. Sustainability 14, 1462. doi: 10.3390/su14031462

[B47] Sarma H. (2011). Metal hyperaccumulation in plants: A review focusing on phytoremediation technology. J. Environ. Sci. Technol. 4, 118–138. doi: 10.3923/jest.2011.118.138

[B48] Seth C. S. (2012). A review on mechanisms of plant tolerance and role of transgenic plants in environmental clean-up. Bot. Rev. 78, 32–62. doi: 10.1007/s12229-011-9092-x

[B49] Shakoor M. B. Ali S. Hameed A. Farid M. Hussain S. Yasmeen T.. (2014). Citric acid improves lead (Pb) phytoextraction in brassica napus l. by mitigating Pb-induced morphological and biochemical damages. Ecotoxicol. Environ. Saf. 109, 38–47. doi: 10.1016/j.ecoenv.2014.07.033 25164201

[B50] Sheoran V. Sheoran A. Poonia P. (2010). Role of hyperaccumulators in phytoextraction of metals from contaminated mining sites: A review. Crit. Rev. Environ. Sci. Technol. 41, 168–214. doi: 10.1080/10643380902718418

[B51] Shevchenko A. Loboda A. Shevchenko A. Ens W. Standing K. G. (2000). MALDI quadrupole time-of-flight mass spectrometry: A powerful tool for proteomic research. Anal. Chem. 72, 2132–2141. doi: 10.1021/ac9913659 10815976

[B52] Sidhu G. P. S. Singh H. P. Batish D. R. Kohli R. K. (2016). Effect of lead on oxidative status, antioxidative response and metal accumulation in coronopus didymus. Plant Physiol. Biochem. 105, 290–296. doi: 10.1016/j.plaphy.2016.05.019 27214085

[B53] Steel R. G. Torrie J. H. Dickey D. A. (1997). Principles and procedures of statistics: A biological approach (McGraw-Hill).

[B54] Tangahu B. V. Sheikh Abdullah S. R. Basri H. Idris M. Anuar N. Mukhlisin M. (2011). A review on heavy metals (As, Pb, and Hg) uptake by plants through phytoremediation. Int. J. Chem. Eng. doi: 10.1155/2011/939161

[B55] Tang L. Hamid Y. Liu D. Shohag M. J. I. Zehra A. He Z.. (2020). Foliar application of zinc and selenium alleviates cadmium and lead toxicity of water spinach–bioavailability/cytotoxicity study with human cell lines. Environ. Int. 145, 106122. doi: 10.1016/j.envint.2020.106122 32950791

[B56] Usman K. Al-Ghouti M. A. Abu-Dieyeh M. H. (2019). The assessment of cadmium, chromium, copper, and nickel tolerance and bioaccumulation by shrub plant tetraena qataranse. Sci. Rep. 9, 5658. doi: 10.1038/s41598-019-42029-9 30948781 PMC6449511

[B57] Venkatachalam P. Jayalakshmi N. Geetha N. Sahi S. V. Sharma N. C. Rene E. R.. (2017). Accumulation efficiency, genotoxicity and antioxidant defense mechanisms in medicinal plant acalypha indica l. under lead stress. Chemosphere 171, 544–553. doi: 10.1016/j.chemosphere.2016.12.092 28039833

[B58] Visioli G. Marmiroli N. (2012). “Proteomics of plant hyperaccumulators,” in Metal toxicity in plants: Perception, signaling and remediation (Springer), 165–186.

[B59] Walliwalagedara C. Atkinson I. van Keulen H. Cutright T. Wei R. (2010). Differential expression of proteins induced by lead in the Dwarf Sunflower Helianthus annuus. Phytochemistry 71 (13), 1460–1465. 20599235 10.1016/j.phytochem.2010.05.018

[B60] Wang Y. Qian Y. Hu H. Xu Y. Zhang H. (2011). Comparative proteomic analysis of cd-responsive proteins in wheat roots. Acta physiologiae. plantarum. 33, 349–357. doi: 10.1007/s11738-010-0554-2

[B61] Wang W. Vignani R. Scali M. Cresti M. (2006). A universal and rapid protocol for protein extraction from recalcitrant plant tissues for proteomic analysis. Electrophoresis 27, 2782–2786. doi: 10.1002/elps.200500722 16732618

[B62] Wang W. Tai F. Chen S. (2008). Optimizing protein extraction from plant tissues for enhanced proteomics analysis. Journal of separation science 31 (11), 2032–2039. 18615819 10.1002/jssc.200800087

[B63] Wang L. Yang H. Liu R. Fan G. (2015). Detoxification strategies and regulation of oxygen production and flowering of platanus acerifolia under lead (Pb) stress by transcriptome analysis. Environ. Sci. Pollut. Res. 22, 12747–12758. doi: 10.1007/s11356-015-4563-y 25913316

[B64] Wu G. Kang H. Zhang X. Shao H. Chu L. Ruan C. (2010). A critical review on the bio-removal of hazardous heavy metals from contaminated soils: Issues, progress, eco-environmental concerns and opportunities. J. Hazard. Mater. 174, 1–8. doi: 10.1016/j.jhazmat.2009.09.113 19864055

[B65] Xian F. Hendrickson C. L. Marshall A. G. (2012). High resolution mass spectrometry. Anal. Chem. 84, 708–719. doi: 10.1021/ac203191t 22263633

[B66] Yurekli F. Kucukbay Z. (2003). Synthesis of phytochelatins in helianthus annuus is enhanced by cadmium nitrate. Acta Bot. Croat. 62, 21–25. https://hrcak.srce.hr/3541

[B67] Zhao Y. Christensen S. K. Fankhauser C. Cashman J. R. Cohen J. D. Weigel D.. (2001). A role for flavin monooxygenase-like enzymes in auxin biosynthesis. Science 291, 306–309. doi: 10.1126/science.291.5502.306 11209081

